# Translational Insights into NK Immunophenotyping: Comparative Surface Marker Analysis and Circulating Immune Cell Profiling in Cancer Immunotherapy

**DOI:** 10.3390/ijms26199547

**Published:** 2025-09-30

**Authors:** Kirill K. Tsyplenkov, Arina A. Belousova, Marina V. Zinovyeva, Irina V. Alekseenko, Victor V. Pleshkan

**Affiliations:** Gene Immunooncotherapy Group, Department of Genomics and Postgenomic Technologies, Shemyakin-Ovchinnikov Institute of Bioorganic Chemistry of the Russian Academy of Sciences, 117997 Moscow, Russia

**Keywords:** cancer immunotherapy, circulating immune cell profiling, immune monitoring, liquid biopsy, mouse models, NK cells, surface markers, translational research

## Abstract

Cells of the innate immune system, particularly natural killer (NK) cells, serve as the first line of defense against tumor development and play a critical role in antitumor immunity. Characterizing the immune cell pool and its functional state is essential for understanding immunotherapy mechanisms and identifying key cellular players. However, defining NK cell populations in mice, the primary model for cancer immunotherapy, is challenging due to strain-specific marker variability and the absence of a universal NK cell marker, such as human CD56. This study evaluates surface markers of NK and other peripheral blood immune cells in both humans and mice, associating these markers with specific functional profiles. Bioinformatic approaches are employed to visualize these markers, enabling rapid immunoprofiling. We explore the translational relevance of these markers in assessing immunotherapy efficacy, including their gene associations, ligand interactions, and interspecies variations. Markers compatible with rapid flow-cytometry-based detection are prioritized to streamline experimental workflows. We propose a standardized immunoprofiling strategy for monitoring systemic immune status and evaluating the effectiveness of immunotherapy in preclinical and clinical settings. This approach facilitates the design of preclinical studies that aim to identify predictive biomarkers for immunotherapy outcomes by monitoring immune status.

## 1. Introduction

Tumors are systemic diseases that affect the entire organism, particularly the immune system. While most research focuses on the immune component within tumors (e.g., tumor-infiltrating lymphocytes, TILs), systemic effects related to the composition and functional status of circulating immune cells may provide not only general insights into the patient’s condition but also serve as a source of biomarkers for assessing disease progression and treatment outcomes. Furthermore, some studies have demonstrated a correlation between immune cell populations in tumors and those in the peripheral blood [1].

Effective antitumor immunotherapy promotes the regression of solid tumors by initiating and sustaining the antitumor immune cycle [2]. We hypothesize that the systemic effects of immunotherapy alter the representation or functional state of specific circulating immune cell subsets. Identification of immune cell populations associated with different treatment responses—whether leading to complete regression or tumor progression—requires analysis of tumor-infiltrating immune cells. However, in cases of complete regression, only circulating immune cells remain accessible for study.

Peripheral blood is an accessible source for immune monitoring and can provide insights into systemic immune status, despite potential differences with tissue-resident immunity. The minimally invasive nature of blood collection enables repeated evaluation of immune cell populations and provides dynamic indicators of therapeutic response. This approach might be particularly valuable in preclinical mouse models, which are foundational to oncology drug development [3,4].

However, a critical limitation of conventional mouse studies is the frequent need for terminal procedures to obtain immune cell data, which prevents longitudinal evaluation of individual animals. Small-volume blood sampling solves this problem by enabling repeated immune profiling without euthanasia. When applied to cancer immunotherapy studies in mouse models, this methodology may reveal correlations between circulating immune signatures and treatment outcomes. We anticipate that these findings can be translated into standardized biomarker panels using conserved markers. This would allow for direct validation in subsequent clinical trials through longitudinal immune monitoring.

This review consolidates reported data on surface markers of circulating T, B, and natural killer (NK) cells, with particular emphasis on mouse NK cells in BALB/c and C57BL/6 strains. NK cells act as the primary line of defense in antitumor immunity due to their ability to recognize and destroy abnormal cells without prior sensitization [5]. Their pivotal role in tumor surveillance goes beyond directly lysing malignant cells. NK cells contribute to multiple stages within the antitumor immunity cycle. They eliminate circulating tumor cells to prevent metastatic spread [6,7,8], and their cytotoxic activity facilitates antigen release. This promotes DC activation and subsequent engagement of the adaptive immune response [9,10]. Additionally, NK cells activate other immune effector cells and sustain an inflammatory microenvironment to help orchestrate a robust and durable antitumor response [9,10,11,12]. Consequently, the functional competence of NK cells is critical to antitumor immunity, and their dysfunction can enable tumor immune evasion. A significant challenge in using NK cells for immunotherapy, especially in strategies that aim to exploit their synergy with T cells, is accurately assessing their in vivo activity.

The cytotoxic function of NK cells depends on various complementary mechanisms that are specific to surface molecules. A key challenge in analyzing mouse NK cells is the absence of a reliable CD56 marker, which is present in humans. This necessitates the use of alternative markers to clearly distinguish NK cells from T cells [13]. Additionally, some mouse NK cell markers are strain-specific. This extends to the expression of defining markers, which can vary substantially between common laboratory mouse strains. For example, the canonical NK cell marker NK1.1 is present in C57BL/6 mice but absent in BALB/c mice. Therefore, other markers, such as using NKp46 or CD49b, should be used to accurately identify NK cells in these strains.

To overcome barriers to species-specific identification, we provide a comparative visualization of conserved and strain-divergent markers for NK cells. This study provides a comprehensive evaluation of surface markers on NK cells and other immune cells in peripheral blood. These markers are characterized by their association with specific functional profiles, and this study explores their translational relevance in assessing the efficacy of immunotherapy interventions. The discussion includes information on the markers, their corresponding genes, ligand interactions, and interspecies variations. The distribution of these markers across various immune cell populations is examined, and targets compatible with rapid, flow-cytometry-based detection are prioritized to streamline experimental workflows. This work emphasizes profiling surface markers on circulating immune cells in tumor diseases because these markers may serve as critical indicators of host immune status during immunotherapy and translational studies. Hematological malignancies are excluded due to their distinct pathophysiology. Other limitations include constraints on antibody specificity, the limited availability of validated reagents, and the deterministic nature of the approach, which precludes the discovery of unpredicted targets. This analysis addresses key methodological challenges in the field, such as significant interspecies marker variability and limitations inherent to current antibody-based profiling, within the broader context of establishing a robust translational bridge between preclinical immune signatures and clinically applicable biomarkers.

## 2. Approaches to Assess the Overall State of the Immune System Using Peripheral Blood

Peripheral blood is used for liquid biopsy due to its minimally invasive collection and systemic representation of tumor burden. The most commonly used analyses include the detection of circulating tumor clusters (CTCs) to elucidate the mechanisms of metastasis and identify novel targets for therapy [14], the measurement of circulating tumor DNA (ctDNA) load to monitor tumor burden and response to treatment, and the use of exosomes as promising biomarkers for diagnosis and prognosis [15]. Due to tumor heterogeneity, ctDNA may be a more accurate representation of the tumor cell population than a biopsy or cytological sample. It is also useful for monitoring treatment resistance [16,17]. Compared to tissue biopsy, peripheral blood analysis better captures tumor dynamics and subclonal CTCs populations.

In the context of the particular interest in blood as one of the liquid biopsies suitable for patient status analysis [18], the implementation of immune cell population analysis in anticancer therapies may be a promising direction of research to determine the effectiveness of therapeutic interventions or in conducting clinical trials.

For an effective immune response, the effector cells of the immune system must be functional and present in sufficient numbers. The main effector cells of the immune system include lymphoid cells, T cells, B cells, and NK cells, as well as some myeloid cells such as neutrophils and monocytes. The first two types of lymphoid cells represent a branch of the adaptive immune response that requires specific recognition of antigens, whereas NK cells belong to innate immunity and can recognize exogenous agents and pathological host cells nonspecifically. Cytotoxic T and NK cells are capable of direct killing of foreign cells [19,20] due to the presence of lytic granules containing perforin (PRF) and granzymes (GZM). Perforin is a pore-forming protein that embeds itself in the target cell membrane, allowing the granzymes to penetrate and induce apoptosis.

The determination of the immune system status by the antitumor immune response index (cytolytic activity, CYT score) was proposed in 2015 [21,22]. This index is calculated based on the expression levels of *GZMA* and *PRF1* mRNA and is associated with markers of cytotoxic T lymphocytes, as well as a positive prognosis for therapy [21]. High cytolytic activity of immune cells indicates their functionality, which has a favorable effect on the outcome of antitumor therapy. This approach is particularly useful in human observational studies.

In general, the characteristics of circulating immune cell populations can be largely dependent on the type of cancer, the stage of progression, and the dynamics of disease development. Monitoring specific fractions of immune cells or combining this with a functional analysis of such cells could provide insight into the state of the body during the treatment of certain tumor diseases.

## 3. Some Examples of Changes in Immune Cell Populations in Cancer

T lymphocytes are often one of the key parameters in immunological monitoring. They tend to decrease in patients with progressive malignancies. Studies have shown significant differences in the representation of various T-cell subpopulations, including CD4^+^ T helpers (Th), regulatory T cells (Tregs), CD8^+^ cytotoxic T lymphocytes (CTL) [23].

The CD4^+^/CD8^+^ cell ratio is genetically determined but it also reflects the status of adaptive immunity at the time of evaluation. With advancing age, cytotoxic T cell populations decrease, while the number of regulatory cells remains stable [24]. In healthy individuals, this ratio typically ranges from 1 to 3 and shows an age-dependent increase in individuals [25]. Reduced ratios may indicate various immunodeficiency disorders or other immune system abnormalities. Mouse models show greater variability, with CD4^+^/CD8^+^ ratios ranging from 1 to 5 depending on strain and age characteristics [26].

In addition to distinct populations of Th and CTLs, circulating CD4^+^CD8^+^ double-positive (DP) T cells represent a highly heterogeneous population associated with pathological conditions [27,28]. DP T cells have been detected in both humans and mice and originate from both CD4^+^ and CD8^+^ T cells. These cells are primarily cytotoxic. However, in mice, some can produce Foxp3 and exhibit suppressive properties. In B16-melanoma mice and in patients with melanoma, non-small-cell lung cancer (NSCLC), breast cancer, or pancreatic cancer, DP T cells accumulate primarily in tumors, where they can constitute up to 3% of all CD3^+^ T cells (compared to <0.5% in peripheral blood) [29]. Compared to healthy donors, patients with urological cancers exhibit higher levels of circulating DP T cells [30]. Contrary to the traditional view that mature DP T cells are rare aberrations arising from thymic selection biases, these cells are present in both tumors and the periphery, maturing in response to disease and environmental stimuli [29,31].

In patients with colorectal cancer (CRC), a decrease in the absolute number of peripheral blood T cells and their subpopulations was observed [32], as well as a decrease in the proportion of INF-γ^+^ cells among total T cells and Th. Patients with advanced CRC had more pronounced signs of immune senescence, manifested by a decrease in the proportion of CD8^+^ naive T cells and CD8^+^ central memory T cells. The proportions and absolute numbers of CD8^+^ and CD4^+^ terminally differentiated effector memory T cells increased, indicating immune senescence. The size of the immune cell populations continues to decrease with tumor progression, which is associated with the immunosuppressive properties of the tumor.

A decrease in circulating CTLs has been reported in patients with primary breast cancer compared to healthy donors [33]. The number of cells recovers after treatment and decreases in relapse.

Studies in NSCLC have demonstrated the prognostic significance of specific circulating Treg populations in the context of anti-PD-1 therapy. High frequencies of circulating Tregs one week after anti-PD-1 therapy correlated with high response rates, longer progression-free survival and overall survival [34].

With regard to NK cell populations, it should be noted that in malignancies, quantitative and functional disturbances in their populations are observed, manifested by a decrease in their cytotoxic activity and a change in the receptor profile. Thus, the activity and number of NK cells may indicate the overall health of the immune system, as described above.

Significantly lower natural killer cell activity was found in healthy individuals with a substantial family history of breast cancer (BC), compared with individuals without a family history of BC [35]. One hypothesis states that certain combinations of KIR molecules and their corresponding HLA ligands that reduce NK cytotoxicity may contribute to the risk of developing BC. It was shown that patients with advanced BC who had combinations of activating KIRs in conjunction with the HLA molecules they recognize (2DS1 + C2 or 3DS1 + Bw4) also had similar inhibitory KIRs (2DL1 and 3DL1, respectively). In contrast, the 2DL1 + C2 and 3DL1 + Bw4 pairs without their activating KIR counterparts were significantly more common in healthy individuals [36]. Bx-haplotype-specific KIRs were elevated in patients with BC. Several other studies have also found an association between breast cancer and B-haplotype specific KIR genes [37,38]. Another study showed that four or more inhibitory KIR genes were associated with a reduced risk of developing cervical cancer [39]. The KIR B-haplotype is thought to be associated with an increased risk of developing solid tumors such as stomach cancer [40]. It is suggested that the role of HLA/KIR combinations may vary significantly in different populations.

In colorectal cancer, quantitative characteristics of cytotoxic NK cells may have prognostic value and provide insight into survival in each individual case. Moderate and high cytotoxic activity of peripheral blood cells correlates with decreased cancer incidence, whereas decreased cytotoxicity is associated with increased cancer risk, suggesting that natural immunologic host defense mechanisms may play a protective role against oncogenesis [41]. A higher percentage of NK cells was associated with longer survival than a lower percentage in colorectal cancer. NK cell percentage was positively correlated with T and B lymphocyte counts and negatively correlated with patient age [42].

B cells, an important component of adaptive immunity, exhibit a variety of different phenotypes during their development and differentiation cycle. Most studies dealing with B cells are related to different hematologic malignancies, which are not discussed here. In the case of solid tumors, different B cell populations are more often considered as tumor-infiltrating immune cells that can have different effects on the tumor depending on the context [43]. It has been reported that in CRC, circulating B lymphocyte populations are elevated compared to those in healthy patients and do not change with tumor growth [32], while there is a decrease in the number of total T cells, Th and CTLs.

The same study showed a decrease in the proportion of IFN-γ^+^ cells among Th and total T lymphocytes [32]. Profiles of cytokines produced by immune cells can also serve as indicators of host immune status [44]. Elevated levels of pro-inflammatory cytokines such as IL-6 and TNF-α may indicate chronic inflammation. A balanced cytokine profile is associated with a healthy immune response.

## 4. Principle Criteria for the Identification of Immune Cells

The primary criteria for immune cell identification are surface markers, which are the most accessible targets for analysis using techniques such as fluorescence-activated cell sorting (FACS). Cell sorting can be used to isolate these populations for in vitro cultivation and functional testing. Although intracellular staining, Western blotting, and PCR can be used to evaluate intracellular markers and assess transcription levels, these methods substantially increase the time and complexity of sample preparation. Furthermore, the cell fixation required for intracellular staining precludes subsequent applications that require viable cells. Consequently, this work focuses primarily on surface markers of immune cells that allow for differentiation of key immune cell populations, particularly in circulating blood.

The primary source of immune cells is the bone marrow, where they develop from a common multipotent primitive cell, the hematopoietic stem cell (HSC) [45,46]. These cells are characterized by the expression of CD45, an evolutionarily conserved Protein Tyrosine Phosphatase Receptor Type C (PTPRC), exclusively present on all nucleated hematopoietic cells except mature erythrocytes and platelets [47]. Therefore, immune cells can be defined as predominantly CD45-positive populations. Peripheral blood, excluding erythrocytes and platelets, represents a circulating pool of these CD45^+^ immune cells that travel throughout the organism to carry out their specialized functions [48].

The cells of the immune system responsible for the immune response are divided into two large categories based on their progenitor cell: lymphoid (T, B, and NK cells) and myeloid (neutrophils, monocytes, eosinophils, and basophils) (see Supplementary Figure S1). In connection with tumor diseases and their control, lymphoid cells are of particular interest, as well as monocytes, which are precursors of dendritic cells (professional antigen-presenting cells, APC) and macrophages, which eliminate pathogenic and foreign cells and organisms. The key characteristics and defining surface markers of these immune cells are outlined below.

### 4.1. T Cells or T Lymphocytes

T cells comprise several subtypes that are distinguished by specific markers and functions. All T cells express the defining marker CD3 [49,50], a protein complex that serves as the primary co-receptor for the T cell receptor (TCR). Cytotoxic T cells express CD8, which binds exclusively to MHC class I and functions as a TCR co-receptor. CD3^+^CD8^+^ T cells mediate the targeted destruction of abnormal and foreign cells. Helper T cells (Th) express CD4, a TCR co-receptor specific for MHC-II, and regulate adaptive immune responses through APCs. The Th population includes functionally distinct subsets (Th1, Th2, Th9, Th17, Th22, etc.) characterized by unique cytokine profiles.

Regulatory T cells (Tregs) are identified by expression of the intracellular transcription factor FOXP3 [51]. These cells maintain self-tolerance under physiological conditions and mediate immunosuppression in cancer.

These markers characterize T cell populations in both humans and mice. Notably, anti-CD3 antibodies can activate the receptor, which should be considered in experimental design.

### 4.2. B Cells or B Lymphocytes

B cells are essential for humoral immunity because they produce antigen-specific immunoglobulins that target and neutralize pathogens. Upon antigen exposure, B cells differentiate into either antibody-producing plasma cells or memory B cells [52]. Memory B cells exhibit rapid reactivation upon re-exposure to their cognate antigen and subsequently differentiate into antibody-secreting plasma cells.

The CD19 marker serves as a universal identifier for B cells in both humans and mice. Distinct developmental stages of B cells are marked by the sequential expression of CD20, CD21, and CD27 [53,54,55,56,57]. These markers maintain consistent expression patterns across human and murine B cells, allowing for their use for population identification in both species [55,58,59].

### 4.3. Monocytes

Monocytes are innate immune cells of the myeloid lineage that circulate in the blood for several days before migrating to sites of inflammation, where they differentiate into macrophages (MFs) or dendritic cells (DCs) [60]. Peripheral absolute monocyte count has been shown to be a valuable diagnostic tool in solid tumor patients. It has been found to be associated with aggressive clinicopathological characteristics and can serve as an independent predictor of unfavorable clinical outcomes [61].

In humans, monocytes express the pattern recognition receptor CD14, which enables bacterial detection through lipopolysaccharide (LPS) binding, and the CD16 receptor involved in antibody-dependent cellular cytotoxicity (ADCC). Based on differential expression of these markers, human monocytes are classified into three subsets: classical (CD14^++^CD16^−^), intermediate (CD14^++^CD16^+^), and non-classical (CD14^dim^CD16^++^) [62].

In mice, monocytes exhibit significantly lower CD14 expression compared to humans and are characterized by Cx3cr1, Ly6C, and CCR2 markers. Mouse monocyte subsets are distinguished by their Ly6C expression levels: classical (Ly6C^high^) and non-classical (Ly6C^low^) [63].

Functionally, classical monocytes in both species primarily mediate inflammatory responses and can differentiate into MFs and DCs [64,65].

#### Macrophages and Dendritic Cells of Peripheral Blood

Macrophages are primarily found in tissues and are usually not present in the blood. They can be maintained by either differentiating from blood monocytes (monocytic origin) or by local proliferation established during embryonic development (resident MFs) [66,67].

F4/80 is a pan-marker of mouse MFs, including those in the liver (Kupffer cells), spleen, peritoneum, and other tissues [68,69]. Humans lack a direct analogue of this marker, and macrophage populations—depending on their origin and polarization—can be characterized by a combination of markers such as CD14, CD68, HLA-DR (MHC class II) and others [70,71,72].

Unlike MFs, dendritic cells are present in small quantities in human blood. The three main DCs subsets can be identified based on their surface marker expression: plasmacytoid DCs (pDCs) CD303^+^CD304^+^ and two types of conventional DCs—CD1^+^ cDCs1 and CD141c^+^ cDCs2 [73,74].

In mice, peripheral blood contains mainly immature DCs (CD11c^+^CD3^−^B220^−^) that later migrate to the spleen where they mature [75].

### 4.4. Neutrophils

Neutrophils, the dominant phagocytic granulocytes of myeloid origin, serve as the first line of defense in the innate immune system. With a circulating half-life of up to 19 h, they mediate rapid responses against extracellular pathogens, including bacteria and fungi. These cells express Integrin αM (CD11b), a pan-myeloid marker conserved in both humans and mice. CD16 and CD66b markers can be used to identify neutrophils in humans [76], and Ly6G in mice [77]. Note that mouse granulocytic myeloid-derived suppressor cells (MDSCs) can also be CD11b^+^Ly6G^+^; so, neutrophils should be distinguished from these cells. High levels of MDSCs in peripheral blood may be negative predictive biomarkers for immune checkpoint therapy [78].

### 4.5. NKT Cells

These cells represent a unique lymphocyte population co-expressing NK cell markers and αβ T-cell receptors (TCRs). Unlike conventional T cells, which exhibit diverse TCR repertoires selected via MHC class I or II, NKT cells possess a restricted TCR repertoire selected by the MHC-like molecule CD1d [79,80]. The CD1d-restricted TCR α-chain is represented by Vα14-Jα18 in mice and Vα24-Jα18 in humans.

Despite their low frequency, NKT cells play crucial roles in immune responses, including tumor surveillance and immune regulation [81].

The prominent invariant NKT cells (iNKT, Type I NKT) have the most specific identification tool in both mice and humans: the αGalCer/CD1d tetramers [82,83]. Alternatively, human iNKT cells can be detected using the 6B11 monoclonal antibody, which targets the conserved CDR3 region of the canonical Vα24-Jα18 chain [84,85]. Notably, defining human NKT cells simply as CD3^+^CD56^+^ is inaccurate [86]. Various options for determining the fraction of NKT cells have been proposed in relevant publications [87,88].

### 4.6. Natural Killer (NK) Cells

Classified as group I innate lymphoid cells (ILCs), NK cells rapidly respond to diverse pathological challenges and display substantial phenotypic and functional heterogeneity, with subsets specialized in cytotoxicity, regulation, and cytokine production. [89,90]. Due to this extreme heterogeneity, defining NK cells has been challenging, and they were originally identified as CD3^−^ IFNγ-producing cytotoxic lymphocytes distinct from T and B cells.

In humans, NK cells are now typically defined as CD3^−^CD56^+^ cells, distinguished by the brightness of the CD56 marker. The CD56^dim^CD16^+^ subset of NK cells is predominantly found in the blood and is highly cytotoxic, while the CD56^bright^CD16^−^ subset is the major subtype found in the lymph nodes and has only weak cytotoxic potential while regulating immunity [90]. However, dividing into functional subtypes based on CD56 expression may not always be accurate. Research has shown that there are intermediate subtypes of CD56^+^ NK cells that exhibit both cytotoxic and regulatory phenotypes [91]. Additionally, populations of CD56-negative NK cells can develop in chronic infections [92,93,94]. Additionally, it has been demonstrated that CD56 expression neither promotes NK cell proliferation nor cytotoxicity nor affects the expression of activating and inhibitory markers [95].

However, mouse NK cells lack a reliable marker equivalent to human CD56, making it difficult to detect these cells and their diverse populations. Instead, combinations of markers such as NK1.1 and CD49b are used to identify mice NK cells, although their utility varies between mouse strains.

Human NK cells express killer cell immunoglobulin-like receptors (KIRs), whereas mouse NK cells predominantly express the Ly49 receptor family. Additional differences exist in the respective maturation stages of NK cells.

Thus, NK cell receptor repertoires differ significantly not only between humans and mice but also between different mouse strains used in preclinical studies. These differences are discussed in detail in the following sections.

## 5. A Brief Outline of Human and Mouse NK Cell Maturation

NK cells originate from CD34^+^ precursors in the bone marrow, where they localize in clustered or dispersed patterns [96,97,98]. Their maturation continues in secondary lymphoid organs, where they differentiate into functional subsets [99,100]. Once mature, NK cells enter the bloodstream. They either remain in circulation or migrate into peripheral tissues, where local factors drive the formation of tissue-resident subtypes [100,101].

**Figure 1:**
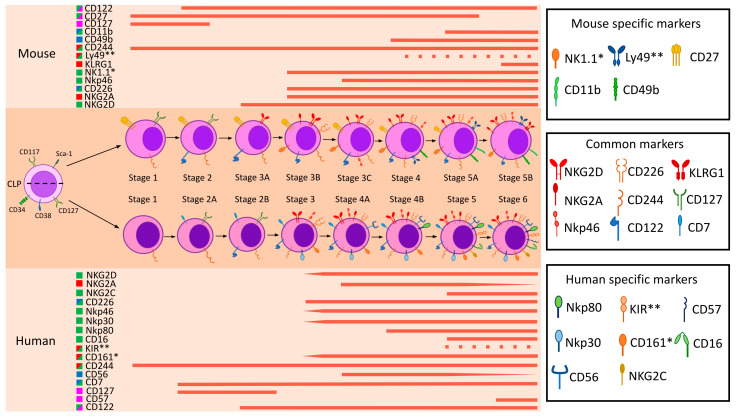
Developmental stages of mouse and human NK cells. The figure shows the expression of markers at different stages of NK cell maturation in humans and mice (C57BL/6 strain). Unique and most represented markers in NK cells are considered as specific markers. The functional characteristics of the markers are shown in different colors: green—activating markers, red—inhibitory markers, blue—cell adhesion and migration markers, pink—differentiation and maturation markers. The dotted lines indicate the expression of receptor families (e.g., KIRs and Ly49s), and the wedge shows the change in expression level of the marker at different maturation stages. *—functionally similar markers in humans and mice are shown (e.g., human CD161 and mouse NK1.1), **—functionally similar receptor families in humans and mice are shown.

NK cell maturation is a tightly regulated selection process that determines their functional competence and ability to elicit effective antitumor and antiviral immune responses. During maturation, NK cells progress through distinct developmental stages, beginning with immature NK cells (iNK) and culminating in mature NK cells (mNK), which exhibit potent cytotoxic activity and the capacity to produce cytokines, including Interferon-γ (IFN-γ) and Tumor Necrosis Factor-α (TNF-α). Mature NK cells are characterized by upregulated expression of activating receptors such as NKG2D and certain Natural Cytotoxicity Receptors (NCRs), as well as enhanced degranulation capacity and elimination of target cells. The key stages of NK cell maturation, as defined by stage-specific markers in humans and mice, are illustrated in Figure 1. A detailed analysis can be found in the relevant publications [102,103,104].

It should be noted that in the tumor microenvironment (TME), NK cell maturation may be impaired [105]. Tumor cells and associated immunosuppressive factors can inhibit the transition of NK cells to a mature state, leading to the accumulation of immature populations with reduced cytotoxic activity and diminished cytokine production. This functional impairment of NK cells represents a key mechanism enabling tumor immune evasion.

## 6. Comparison of Human and Mouse Surface NK Markers

The functional activity of NK cells is determined by the balance of activating and inhibitory signals transmitted by specialized surface receptors and their co-stimulatory/inhibitory molecules. The interplay between these signals determines the overall responsiveness of NK cells to tumor cells, highlighting the importance of understanding these mechanisms for developing effective immunotherapeutic strategies.

Activating receptors predominantly signal through **immunoreceptor tyrosine-based activation motifs (ITAMs)**. Ligand binding induces ITAM phosphorylation by Src family kinases, initiating downstream events including calcium mobilization, cytotoxic granule release, and cytokine production [106]. Inhibitory receptors mostly employ **immunoreceptor tyrosine-based inhibitory motifs (ITIMs)**, which recruit phosphatases that dephosphorylate multiple targets, inhibiting NK cell function. In tumor diseases, this balance is often disrupted, suppressing NK cell activity due to immunosuppressive factors in the TME [11].

**Figure 2:**
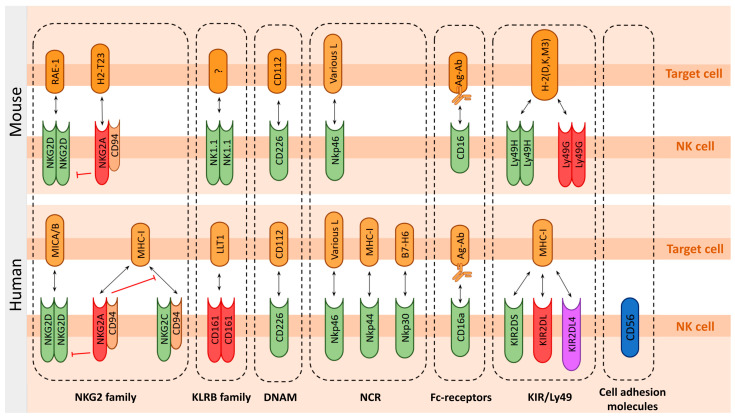
Characteristic receptors of NK cells and their ligands. The figure shows the receptors and ligands of human and mouse NK cells. Only the main receptors under consideration are shown to have the possibility of multimerization. Any adapter proteins are omitted. Activating receptors are green, inhibitory receptors are red, receptors with activating and inhibitory properties are purple, and cell adhesion molecules are blue. Ligands are orange. Interactions between NKG2 family receptors are also depicted.

To assess the functionality of the immune system and its potential response to tumors, the distinction of NK cell receptors into activating and inhibitory types is more relevant than their structural subdivision. For example, Killer cell Immunoglobulin-like Receptors (KIRs) in humans can be activating and inhibitory or, in the case of the highly conserved KIR2DL4 in primates, combine both functions [107,108,109]. Therefore, we will focus primarily on the activating receptors responsible for the antitumor functions of NK cells. We will review the most prominent groups of NK cell receptors and compare how they differ between humans and various mouse strains. A graphical representation of these molecules and their interactions is shown in Figure 2.

### 6.1. Function-Determining Molecules of the NK Cells

#### 6.1.1. Death Ligands in NK Cell-Mediated Cytotoxicity

The Tumor Necrosis Factor (TNF) superfamily members TRAIL (TNF-related apoptosis-inducing ligand) and FasL (CD95L) are key death ligands involved in NK and T cell function. These ligands induce apoptosis through the extrinsic pathway by binding their respective death receptors (TRAIL R1/R2 and Fas), which triggers caspase activation directly at the cell membrane [5]. This mechanism is distinct from the intrinsic, mitochondrial-mediated apoptosis triggered by cytotoxic granule components like granzymes.

#### 6.1.2. Lectins and Lectin-like Receptors

Immune surveillance by innate immune cells is regulated by a diverse repertoire of conserved Pattern Recognition Receptors (PRRs). These receptors recognize Pathogen-Associated Molecular Patterns (PAMPs) or Damage-Associated Molecular Patterns (DAMPs) from dead and dying cells [110,111] and function as scavenger receptors, sensors of cell death and transformation, and regulators of immune homeostasis [112]. PRRs are typically classified by domain architecture, phylogeny, function, or cellular localization.

They include C-type lectin receptors (CLECs) and related C-type lectin-like receptors (CTLRs). Canonical CLECs contain a C-type lectin-like domain (CTLD) and require Ca^2+^ for ligand binding, whereas CTLRs lack this ion dependence. However, due to incomplete structural characterization of many binding domains [113], the distinction between CLECs and CTLRs remains challenging. Consequently, some receptors have been reclassified over time, leading to inconsistencies in the literature where the same receptor may be labeled differently depending on the publication date [112].

##### NKG2 Family

The NKG2 family of proteins functions as receptors within complex receptor-ligand signaling networks, exhibiting both inhibitory and activating properties. The NKG2 proteins are C-type lectins-like receptors [114], some of which can dimerize with CD94 to form heterodimers. NKG2 receptors are expressed primarily on NK cells and CD8^+^ T cell subsets, with some members also found on γδ T cells, NKT cells, certain CD4^+^ T cell subsets, and myeloid cells. Genetically, NKG2A is encoded by *KLRC1*, NKG2D by *KLRK1*, and NKG2C by *KLRC2* in humans.

The **NKG2D** forms a homodimer and is a major NK cell activating receptor. It binds to several ligands, some of which are largely referred to as “stress ligands”, such as MHC class I polypeptide-related sequence A and B (MICA/B) and the UL16-binding proteins (ULBPs) molecules family in humans [115]. In mice, Retinoic acid early inducible genes (RAE-1 α-ε glycoproteins) define a ligand family for the activating NKG2D receptor [116]. Two RAE-1 proteins are encoded by the *Raet1d* and *Raet1e* genes in C57BL/6 mice. Three other *Raet1* genes (*Raet1a-c*) have been identified in various mouse strains, but they are not present in C57BL/6 [117].

In humans, NKG2D is expressed by almost all NK cells, and some subsets of T cells such as NKT, γδ T cells, CD4^+^ T and CD8^+^αβ T cells [118]. In mice, NKG2D is expressed by NK cells and γδ T cells, as well by activated CD8^+^αβ T cells and macrophages [119].

**NKG2A** is an inhibitory receptor that represents an attractive target for immune checkpoint inhibition (ICI) through therapeutic antibodies [120]. This receptor forms a heterodimer with CD94 and recognizes non-classical MHC class I molecules, specifically HLA-E in humans [121]. The functional murine homolog of HLA-E is Qa-1b (encoded by H2-T23) [122].

HLA-E is expressed in most human tissues, although at levels approximately 25-fold lower than those of classical MHC class I molecules [120]. HLA-E plays a specialized role in NK cell recognition by presenting peptides derived from the signal sequences of classical MHC class I molecules (HLA-A, -B, -C, and -G). Interaction of the NKG2A-CD94 heterodimer with peptide-loaded HLA-E triggers phosphorylation of the ITIMs in NKG2A, which recruits the phosphatase SHP-1. SHP-1 then suppresses activating signals from both T cell receptor ITAMs and receptors such as NKG2D, thereby inhibiting cytotoxic responses. Therapeutic antibodies targeting NKG2A can block this inhibitory signaling, allowing activation by NKG2D or TCR to restore tumor cell killing [123].

**NKG2C** is an activating receptor in humans, forming a heterodimer with CD94 (NKG2C/CD94). The activating NKG2C/CD94 and inhibitory NKG2A/CD94 receptors bind competitively to their common ligand, HLA-E, with the inhibitory receptor exhibiting approximately six-fold greater binding affinity. Basal levels of HLA-E activate NKG2A^+^ NK cells without significantly stimulating NKG2C^+^ NK cells, thereby maintaining a balance in NK cell reactivity [124].

NKG2A and NKG2C receptors are not co-expressed on peripheral CD56^dim^ NK cells. However, almost all peripheral CD56^bright^ NK cells with active NKG2C co-express NKG2A [125,126].

Specifically, NKG2C^+^ NK cells have enhanced cytolytic activity and can target HLA-E-expressing tumors, including certain glioblastomas [127,128]. NKG2C has emerged as a potential biomarker for assessing human cytomegalovirus (HCMV) control and predicting vaccination response [124,129,130]. This infection alters the frequency of NKG2C^+^ NK cells, which should be taken into account [131,132]. Individuals with a robust vaccination response have increased numbers of CD56^dim^CD16^+^NKG2C^+^ peripheral NK cells compared to those with a weaker response [130].

Despite the expression of *Klrc2* transcripts in C57BL/6j mouse NK cells (CD3^−^NK1.1^+^Nkp46^+^), the corresponding NKG2C receptor was not detected in mice [133]. Therefore, NKG2C is unlikely to have translational potential.

##### NKRP1 Family—Human KLRB1 vs. Mice NK1.1 Receptors

The NKRP1 family has a very complex and confusing history of classifications, overlapping names, and problems in domain structure determination [134]. The structural similarities of the human NKR-P1A receptor and related murine receptors, as well as their ligands, are proposed to vary and sometimes change as new information is obtained [134,135,136].

For our purposes, it is important to note one fundamental point: NKRP1A, also known as CD161, encoded by the *KLRB1* gene, is the only human homolog of the rodent NKRP1 family. In mice, the NKRP1 family contains a set of activating and inhibitory receptors represented by numerous genes and their respective ligands (recently reviewed in [112]).

In humans, KLRB1 (killer cell lectin-like receptor subfamily B, member 1) most likely plays an inhibitory role on NK cells [137,138]. In humans, CD161 is expressed on NK, CD4^+^ and CD8^+^ T cells. T cell activation increases CD161 expression [139,140,141]. In NK cells, CD161 interacts with LLT1, which decreases cytotoxicity and granzyme B production [142,143].

In mice, the NK1.1 (*Klrb1c*) surface antigen is also known as CD161b/CD161c/Ly-55. It is in some mouse strains, including C57BL/6, FVB/N, and NZB, but not in BALB/c, CBA/J, C3H, DBA/1, DBA/2 and NOD [144,145]. Why NK1.1 is not detected in some mouse strains is discussed elsewhere [146]. In mice, NK1.1 is expressed on NK cells and some CD4^+^ T cell populations, such as Tregs [147,148,149]. In NK cells, NK1.1 plays a critical role in activation, IFN-γ production, and cytotoxic responses against tumor and infected cells [145,147]. Its expression correlates with tumor cell lysis in vitro, bone marrow allograft rejection in vivo, and the release of cytotoxic granules [150,151,152].

#### 6.1.3. DNAM-1 (CD226)

DNAM-1, an immunoglobulin-like transmembrane glycoprotein, is another major NK cell-activating receptor and, together with NKG2D and some NCRs, contributes significantly to mediating the killing of tumor or virus-infected cells by binding specific ligands. DNAM-1 specifically recognizes PVR (CD155) and Nectin-2 (CD112) ligands, which are expressed on some virus-infected cells and on a broad spectrum of tumor cells of both hematological and solid malignancies [153]. In humans, DNAM-1 is stably expressed on NK and T cells [154], while expression in mice is heterogeneous and correlates with Ly49H/Ly49D receptor levels [155,156].

#### 6.1.4. Natural Cytotoxic Receptors: NCR Family

The NCR family includes the type I transmembrane receptors NKp30 (encoded by the *NCR3* gene), NKp44 (*NCR2*), and NKp46 (*NCR1*) [157,158]. NCRs contain charged residues in their transmembrane regions for association with ITAM-bearing signaling polypeptides: DAP12 for NKp44 and ζ–γ for NKp46 and NKp30 [159].

NKp44 expression is primarily restricted to primates, whereas NKp30 is also present on rat NK cells [160]. Both NKp30 and NKp44 are specific markers for human NK cells [158], but are not expressed in mice (Figure 2). The mouse *NCR3* gene encoding NKp30 is generally a pseudogene [161], except in *Mus caroli* where it remains functional [162]. In contrast, NKp44 is completely absent in mice [162].

NKp46 is evolutionarily conserved across mammals, making it the most suitable marker to identify NK cells in different species and to identify a novel NK cell subset [163,164]. However, in mice, NKp46 can also be detected on rare T cell subsets [165,166].

#### 6.1.5. CD16 (FcγRIII) IgG Receptor

CD16, a low-affinity IgG receptor, is expressed on large granular lymphocytes of both NK and T cell lineages and plays a key role in antibody-dependent cellular cytotoxicity. In peripheral blood, approximately 15–20% of lymphocytes express CD16, whereas its expression in bone marrow lymphocytes is significantly lower (<5%) [167]. In the solid tumor microenvironment, CD16 expression can be reduced by TGF-β, limiting the effectiveness of ADCC [168].

IgG facilitates pathogen opsonization, enabling targeted immune destruction. Its antigen-binding fragments (Fabs) confer specificity, while the crystallizable fragment (Fc) engages Fc γ receptors (FcγRs) on innate immune effector cells, linking adaptive and innate immunity.

Fc receptor III (FcγRIII), known as CD16, is the major mediator of ADCC and plays a critical role in tumor clearance [169,170]. Despite high structural and sequence homology, the two isoforms—CD16a (transmembrane) and CD16b (GPI-anchored)—exhibit markedly different affinities for IgG1, with CD16a binding ~10-fold more tightly due to a single residue difference (Gly-129 vs. Asp-129) [171]. CD16a is sensitive to N-glycan composition, while CD16b is less affected. This difference underscores the functional specialization of these receptors in immune effector responses. In humans, CD16a is expressed mainly on NK cells and to a lesser extent on macrophages and monocytes [172]. CD16b is expressed mainly on neutrophils [171].

Mature human NK cells constitutively express high levels of CD16A (FcγRIIIA), which is considered the most potent inducer of degranulation [170,172]. Mouse NK cells CD16 (FcγRIII), which shares greater homology with human CD32A (FcγRIIA)—a receptor that is absent on human NK cells [173,174,175]. The functional ortholog of human CD16A in mice, CD16-2 (FcγRIV), is absent on resting NK cells [176,177] and uniquely binds IgE, driving IgE-mediated inflammation [178,179]. Unlike human CD16A, neither mouse CD16 nor CD16-2 undergoes ADAM17-mediated shedding [170,180,181], highlighting divergent regulatory mechanisms.

It should also be noted that due to its immunoglobulin binding properties, the CD16 receptor is typically masked by an Fc-block in many marker assays to avoid signal blurring and requires the use of special protocols.

#### 6.1.6. KIRs and Ly49—Mutually Exclusive Human and Mouse Receptor Families

The receptor families—Killer cell Immunoglobulin-like Receptors (KIRs) in humans and Ly49 in mice—interact with major histocompatibility complex (MHC) molecules on target cells. Although functionally analogous in regulating NK cell activity through MHC recognition, these receptor families exhibit structural divergence: Ly49 receptors belong to the C-type lectin family (type II transmembrane proteins), whereas KIRs are immunoglobulin-like receptors (type I transmembrane proteins). Both families include inhibitory and activating members that modulate NK cell function through MHC interactions.

KIRs recognize HLA molecules, including non-classical MHC I variants, whereas Ly49 specifically binds H-2 complex molecules in mice. These receptor families exhibit pronounced polymorphism and polygenicity, reflecting their evolutionary adaptation to pathogen diversity. Both receptor families contribute to NK cell maturation [182,183], immune surveillance and antiviral defense [184].

##### Killer Cell Immunoglobulin-like Receptors (KIRs) Family

KIRs are a family of human and primate activating and inhibitory receptors that are predominantly expressed on NK cells and late-stage differentiated T cell subsets [185].

KIRs are classified based on their structural and functional properties. By number and structure of extracellular immunoglobulin domains (D). By the nature of the intracellular signaling domains—inhibitory KIRs with long (L) cytoplasmic tails containing ITIMs or activating KIRs with short (S) cytoplasmic tails. The latter lack immunoreceptor motifs but are associated with adaptor molecules, usually DAP12, that contain activating ITAM motifs. Fourteen KIRs have been identified that induce either inhibition (3DL1-3, 2DL1-3, 2DL5) or activation (3DS1, 2DS1-5) or both (2DL4) [36]. For details on KIRs and their ligands, see ref. [186]. KIRs are usually located on the cell surface, with the exception of KIR2DL4, which is not detectable on the surface of primary resting NK cells isolated from peripheral blood [187].

##### Ly49 Family Receptors

The Ly49 family of receptors are homodimeric glycoproteins encoded by a highly polymorphic *Klra* gene cluster. The ligands of the Ly49 receptors are both classical MHC-Ia molecules (H-2D and H-2K) [188,189,190] and non-classical MHC-Ib (H2-M3), defined as a ligand for the inhibitory Ly49A [191]. Comparative analysis reveals distinct Ly49 family gene expression profiles between BALB/c and C57BL/6 mouse strains. Similar to human KIRs, they are expressed on both NK and T cells of mice [184]. The activating receptors of the Ly49 family are Ly49 (D, H, L), the inhibitory receptors are Ly49 (A, B, C, E, F, G, I, J, Q) [184]. In BALB/c mice, the *Klra* gene cluster contains six functional genes and two pseudogenes; this cluster lacks the regions encoding the Ly49H and Ly49d receptors [192]. C57BL/6 mice have a broader repertoire of Ly49 family genes and, unlike BALB/c mice, have the activating receptors Ly49H and Ly49D [193]. The major inhibitory receptor of the Ly49 family in C57BL/6 is Ly49C [194].

We used Ly49G for our analysis because it is present in both the BALB/c and C57BL/6 mouse strains, and evidence of this marker’s expression at the protein level has been reported [195,196]). However, differences in the structure of Ly49G ectodomains between these strains should be taken into account when selecting antibodies [197].

### 6.2. Degranulation Markers

A hallmark of NK cell activation is degranulation [198]. NK cell degranulation involves the release of lytic granule contents such as perforin and granzymes that induce apoptosis of target cells. CD107a (lysosome-associated membrane protein 1, LAMP-1) is initially localized to the inner surface of lytic granules and is exposed on the NK cell membrane after exocytosis, preventing pore formation and protecting them from perforin-induced damage. Surface expression of CD107a/LAMP-1 may represent a general mechanism for temporarily limiting cytotoxic cell self-destruction during target cell killing [199]. CD107a externalization has been shown to be a marker of degranulation in NK cells [200,201], CD8^+^ T cells [202], and mature CMV-specific CD4^+^ T cells [203]. Intracellular expression of CD107a/LAMP-1 correlates with lytic granule content and surface expression reduces perforin binding. Knockout of CD107a/LAMP-1 in primary human NK cells and its deficiency in mice resulted in increased NK cell apoptosis upon target cell-induced degranulation [199].

### 6.3. Other Characteristic Markers of NK Cell

Beyond the activating and inhibitory receptors that regulate NK cell function, adhesion molecules mediate critical cell–cell interactions during immune responses. Some adhesion molecules, such as ICAM-1 (essential for immune synapse formation) and CD44 [204,205], are broadly expressed in multiple cell types. Others, such as human CD56 or CD49b in mice, are usually restricted to NK cells.

**CD56** in human blood is strongly associated with natural killer cells, whereas mouse NK cells lack this marker [206]. However, it should be noted that CD56 (Neural cell adhesion molecule—NCAM) is often considered a marker of neural lineage commitment due to its discovery site [207].

**CD49b** (Integrin Subunit Alpha 2, encoded by *ITGA2* gene) is a part of the heterodimeric α2β1 integrin expressed in both humans and mice. CD49b is expressed on NK cells, Tregs, CD4^+^ and CD8^+^ T cells [208,209,210]. CD49b expression on NK cells increases their collagen adhesion, cytotoxic properties and IFN-γ production [211,212]. Thus, CD49b is not a strictly specific marker for NK cells, but can be used to identify them. CD49b may be a reliable marker to identify circulating NK cells in mice [213], whereas CD49b expression on circulating NK cells is weaker in humans [214].

**Ly6a** (Sca-1, Stem Cell Antigen 1) is a general marker for mouse stem cells and some immune cells. Sca-1 is used to identify mice hematopoietic stem cell populations [215]. In the regard of NK cells, it can serve as a marker for early, non-selective NK cell activation [216]. C57BL/6 mouse strain CD3^−^NK1.1^+^Nkp46^+^Sca-1^+^ NK cells have been shown to express higher levels of IFN-γ than Sca-1^−^ NK cells [216].

In mice, the Ly6 family is highly abundant and located in syntenic genomic loci on chromosome 15. Until recently, no *LY6* genes have been annotated at the corresponding human locus. However, LY6S—a solitary human *LY6* gene expressed by spleen cells-has been identified. It shares common ancestry with the murine Ly6a subfamily gene cluster [217].

**CD69**, a membrane-bound C-type lectin-like receptor [218,219] (or referred as type II C-lectin receptor [220]), was not included among major activating markers due to its incompletely understood function. Early studies identified CD69 as one of the first markers upregulated during NK cell activation [221], highlighting its sensitivity to immune stimulation. CD69 is absent on resting NK cells, but is rapidly induced upon activation by cytokines and CD16 antigen binding or TCR/CD3 engagement in T cells [216,222]. Upon activation, CD69 is expressed on human (CD3^−^CD56^+^) [223,224] and mouse (CD11b^+^CD27^+^Nkp46^+^) [225] blood NK cells.

While it is widely used as a marker of early activation of lymphocytes and NK cells [226], as well as a variety of activated lymphocytes, including Th17 cells and a subset of Tregs, underscoring its complex role in immune responses and inflammation often associated with autoimmune diseases, its role remains ambiguous. Some studies associate it with cytokine release as well as homing and migration of activated lymphocytes [220].

Human and mouse CD69 share similar structural features, including the C-type lectin-like domain fold and dimerization interface. Key differences include sequence variations in the putative ligand binding site and minor structural divergences, which may affect ligand specificity and homodimer stability. Both lack the Ca^2+^ binding sites typical of true C-type lectins [218].

**Figure 3:**
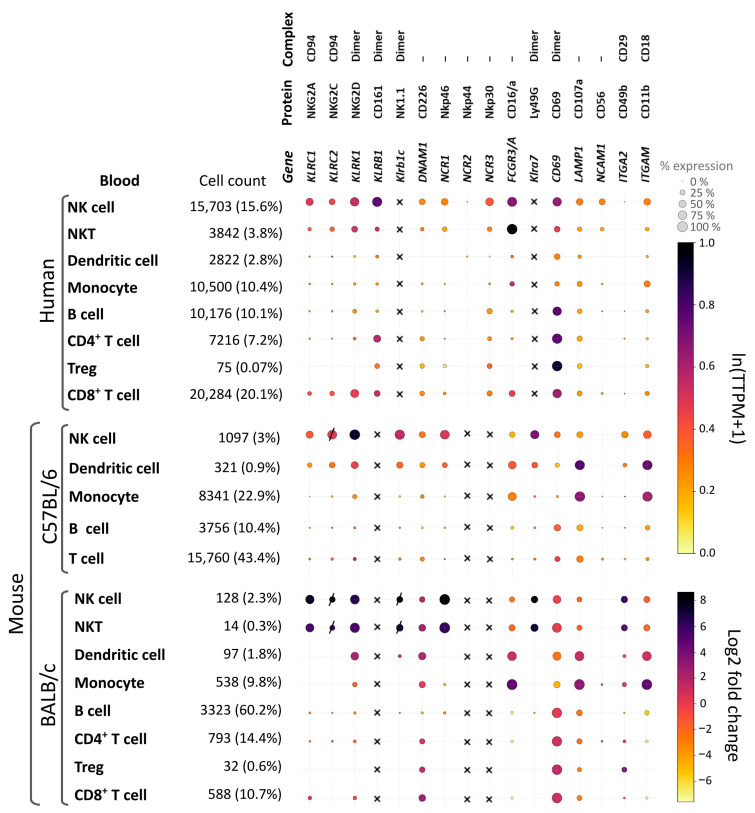
Expression of NK cell markers in humans and mice (C57BL/6J and BALB/c strains). The image depicts a dot plot diagram illustrating the expression of markers in NK cells, and their representation on other types of immune cells. The color of the dot indicates the level of gene transcription, and the size is the percentage of cells expressing that marker. A dashed dot indicates that the gene is expressed only at the transcript level/no Ab binding. Crosses indicate the absence of the marker gene in that species/strain. For humans and C57BL/6, the percentage of cells in the blood was calculated relative to the number of total PBMC, for BALB/c relative to the number of annotated cells in the dataset. The marker expression data were obtained from the specified datasets. The datasets for Human [227] and C57BL/6 [228] marker expression data were taken from CZ CELLxGENE Discover [229]. Blood marker expression data in BALB/c were taken from the PBMCs from BALB/c mice—5′ gene expression, Universal 5′ Gene Expression dataset analyzed using Cell Ranger v3.0.0, 10x Genomics (19 November 2018). Clusters were annotated manually using 10x Genomics Loupe Browser v8.1.2. All dot plots were created using the Matplotlib (3.10.0) Python (3.12.11) library.

Figure 3 summarizes the expression profiles of the markers used to distinguish NK cells from other peripheral blood immune cells and evaluate their functional states. The figure also provides a cross-species analysis of these markers in humans and certain mouse strains. Relative transcription levels were quantified using computational bioinformatics tools applied to publicly available datasets.

### 6.4. Systematic Comparison of Key NK Cell Surface Markers to Access Their Translational Relevance

Effectively translating NK cell research from mouse models to humans requires an understanding of the similarities and differences in immunophenotyping. We provide a comparative analysis of the main surface markers in humans and in common mouse strains (C57BL/6 and BALB/c) (see Supplementary Table S1). This comparison links phenotypic markers to functional states, highlights subset-specific expression, and outlines critical limitations for translational research.

This comparative analysis highlights that, although the fundamental paradigms of NK cell biology—cytotoxicity, activation, and inhibition—are conserved, the specific tools used to evaluate them are not. The absence of a universal marker such as human CD56 in mice requires a multi-parameter, strain-specific approach in preclinical studies. To ensure translational success, the focus should shift toward functionally conserved markers (e.g., NKG2D and CD107a) and conserved functional pathways (e.g., ADCC via CD16 and inhibition via NKG2A) rather than relying on direct phenotypic homologs. Prioritizing these conserved elements in immune monitoring strategies will ensure that insights gained from mouse models are robust, reproducible, and directly relevant to the development and clinical assessment of NK-cell-targeting immunotherapies.

## 7. Approaches to the Assessment of Host Immune Status by Analysis of Cell Populations

We propose that immunotherapy-induced systemic changes in immune cell subsets, which are detectable by blood analysis in preclinical models, may correlate with treatment response (e.g., tumor regression vs. progression). If validated, these immune profiles could serve two purposes: (1) providing insight into therapeutic efficacy and (2) enabling translation into clinical research through the development of standardized cross-species biomarkers.

Immunotherapy exerts its effects systemically; so, monitoring changes in immune cell subsets is essential to evaluating treatment efficacy. It has been proposed that immunoprofiling is best performed using flow cytometry-based methods, either via the classical approach with discrete fluorophores or with multiplexed assays. This assumption is based on the rapid advancement of microfluidic and detection technologies. Notably, spectral flow cytometry has significantly improved the discrimination of spectrally similar fluorophores, overcoming a major previous limitation. This technological progress is accompanied by simplified operator requirements, as most cell separation and analysis steps can now be automated, enhancing reproducibility and throughput. Flow cytometry is already being used to study hematological malignancies, and it is anticipated that it will soon become routine practice [230]. In this context, minimally invasive analyses of small blood volumes enable longitudinal immunoprofiling, poised to become the standard for dynamic monitoring.

A clinically relevant immunoprofiling foundation can be established during the preclinical stage by identifying marker panels that distinguish responders from non-responders. To translate findings from preclinical to clinical settings, marker panels should be designed based on the mechanism of action of investigational therapeutics and their presumed impact on specific immune cell populations. For instance, if natural killer (NK) cells are the targeted population, the immunoprofiling strategy should capture relevant changes in their frequency and/or functional state. When profiling NK cells to assess immune status, the focus should be on their activating receptors to distinguish them clearly from other immune populations. Priority should be given to conserved species-specific markers with harmonized activation markers to ensure translational relevance. Due to their pivotal role in innate antitumor immunity, the analysis of NK cells should focus on cytotoxicity-triggering receptors (cytotoxic signatures). This approach may reveal correlations between NK cell activation and therapeutic efficacy, providing valuable insights for immunotherapy development.

In certain scenarios, changes in immune cell pools may be undetectable using a surface-marker-based panel [231]. In such cases, in addition to refining the marker panel, alternative technologies, such as Next-Generation Sequencing (NGS), can be used for further investigation. However, these methods are guided by principles that are distinct from those of flow cytometry, and they are not yet considered routine for rapid clinical monitoring.

A standardized immunoprofiling workflow is essential to ensure translational relevance and data comparability. This approach should integrate surface marker analysis with functional assessment. The following requirements should be met:

- **Standardized immunoprofiling** is essential for translational relevance. Prioritizing conserved surface markers across species in a workflow enables the direct correlation of preclinical findings with clinical outcomes.
- **NK Cell Identification**: In mouse models, NK cells are usually identified as CD3^−^NK1.1^+^ in the C57BL/6 strain and as CD3^−^Nkp46^+^ in the BALB/c strain. Additional markers, such as CD49b, are sometimes used. In humans, NK cells are identified as CD3^−^CD56^+^ populations. Accurate analysis requires careful exclusion of T and B cell populations.
- **Priority to Activating Markers**: The primary markers of interest are NKG2D, CD107a, and CD69 because they indicate activation status and functional potential. An alternative approach is to examine inhibitory receptors (NKG2A and PD-1) to identify functionally suppressed NK cell subsets that may affect treatment outcomes.
- **Longitudinal monitoring** of circulating immune cells provides a dynamic readout of therapy efficacy. This approach captures systemic immune activation, distinguishes responders from nonresponders, and aligns with the principles of minimally invasive liquid biopsy.
- **A phased translational pipeline**—from mechanistic validation in murine models to biomarker confirmation in clinical trials—is critical for developing predictive signatures. This strategy ensures that immune profiles are biologically grounded and clinically actionable for patient stratification.

Adopting this standardized approach can improve the reproducibility of immune monitoring and accelerate the development of effective NK cell-based immunotherapies.

## 8. Conclusions and Future Perspectives

A combined strategy for NK cell immunoprofiling to assess immunotherapy efficacy should focus on activating receptors as this provides critical insights into antitumor immunity. This approach facilitates the identification of correlations between NK cell activation and therapeutic outcomes, especially in cancer immunotherapies that engage NK cells.

A standardized NK cell immunoprofiling approach could provide a robust framework for assessing immune status in cancer immunotherapy research. This approach integrates immunophenotypic characterization, functional testing, and transcriptomic analysis to ensure reliable and reproducible translation of data from preclinical studies to clinical applications. This strategy is particularly valuable for identifying potential responders and optimizing treatment protocols in different research phases.

The primary challenge in this field is the significant variability in markers between species and mouse strains, which can obscure insights into translation. Our proposed workflow addresses this issue by prioritizing a core set of functionally conserved markers (e.g., NKG2D, CD107a, and CD69) over sequence-homologous markers. This strategy ensures that the biological signals being measured—activation, cytotoxicity, and exhaustion—are comparable across species and strains.

The critical next step is the rigorous application of this stratified approach. First, we should define immune signatures that correlate with treatment response in well-controlled preclinical models using strain-appropriate panels. Then, we can identify the most robust and conserved biological events. Next, we should deliberately validate these specific signatures in clinical trials using the harmonized human counterpart of the core panel. This closed-loop process moves from mouse to human or human to mouse and ensures that the resulting biomarkers are genuine indicators of a therapeutic immune response and not artifacts of a specific model system.

We should focus on the biological meaning of the markers we choose. By anchoring our assays to conserved functions and clear translational pipelines, we can overcome the variability inherent to immunophenotyping. This allows us to generate reproducible and comparable data that ultimately predicts clinical outcomes.

## Data Availability

The data presented here is available throughout this article and in Supplementary Figure S1 and Table S1.

## Supplementary Materials

The following supporting information can be downloaded at: https://www.mdpi.com/article/10.3390/ijms26199547/s1.

## Abbreviations

The following abbreviations are used in this manuscript:

ADCC	antibody-dependent cellular cytotoxicity
BC	breast cancer
ctDNA	circulating tumor DNA
CTCs	circulating tumor clusters
CTL	CD8^+^ cytotoxic T lymphocyte
CLEC	C-type lectin receptor
CTLR	C-type lectin-like receptor
CTLD	C-type lectin-like domain
CRC	colorectal cancer
DC	dendritic cell
DP T cell	double-positive T cell
GZM	granzyme
HSC	hematopoietic stem cell
ITAM	immunoreceptor tyrosine-based activation motif
ITIM	immunoreceptor tyrosine-based inhibitory motifs
KIR	killer cell immunoglobulin-like receptor
LPS	lipopolysaccharide
MHC	major histocompatibility complex
MF	macrophage
NCR	natural cytotoxicity receptors
NK	natural killer
NSCLC	non-small-cell lung cancer
PBMC	peripheral blood mononuclear cell
PRF	perforin
TCR	T cell receptor
Th	CD4^+^ T helper
TME	tumor microenvironment
Treg	regulatory T cell
