# Supplementary material for: Translational Insights into NK Immunophenotyping: Comparative Surface Marker Analysis and Circulating Immune Cell Profiling in Cancer Immunotherapy
Source: Int J Mol Sci. 2025 Sep 30;26(19):9547. doi: 10.3390/ijms26199547 (PMC12524738; doi:10.3390/ijms26199547)
Supplement: Supplementary file 1 [file ijms-26-09547-s001.zip › Figure S1. NK Supplementary Immune markers.pdf]

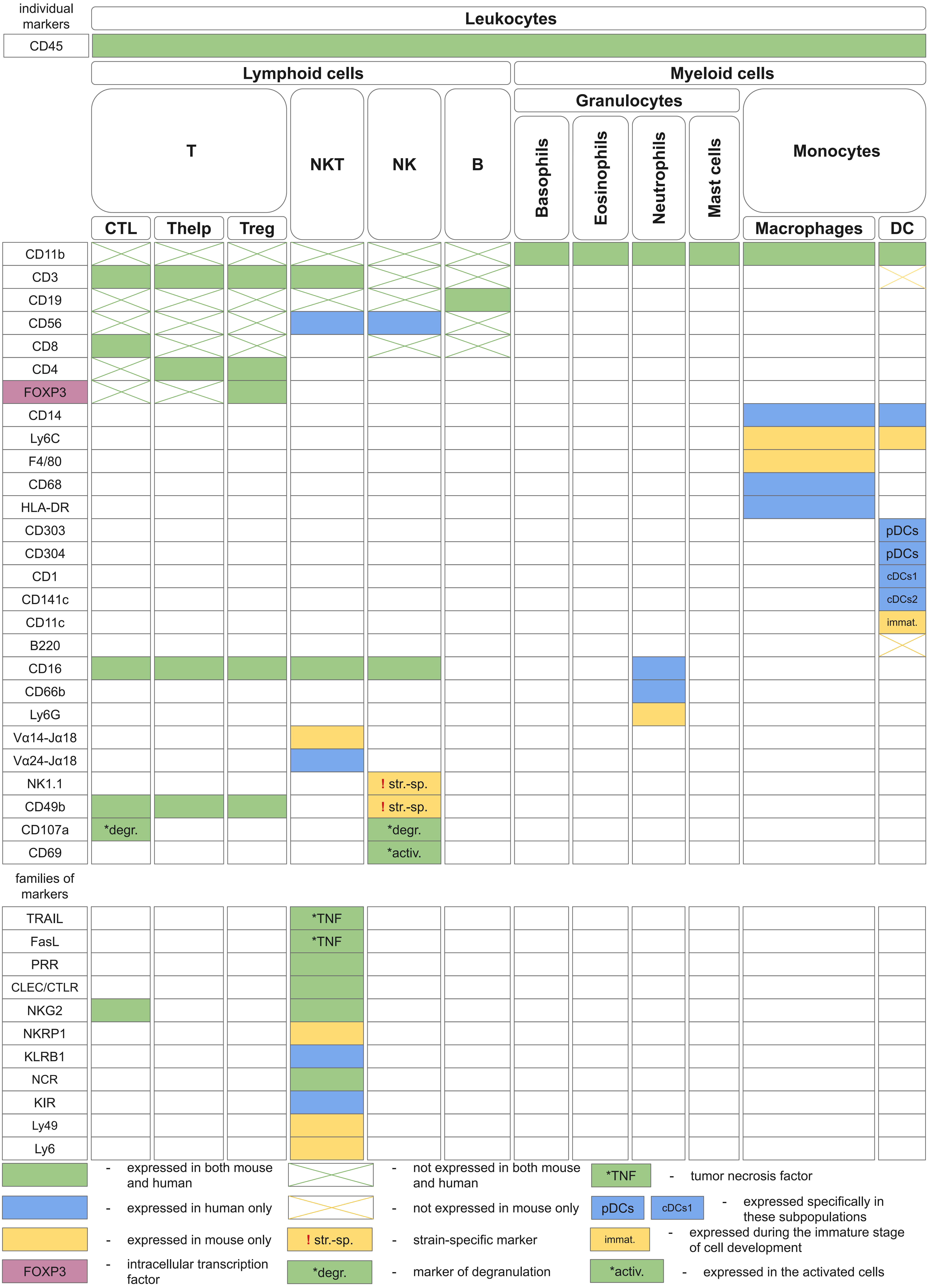

Supplementary Figure S1. Expression patterns of individual immune cell surface markers across various leukocyte subsets, including lymphoid and myeloid lineages. **Lymphoid populations** include cytotoxic T lymphocytes (CTL), T helper (Thelp) cells, regulatory T cells (Treg), natural killer T cells (NKT), natural killer cells (NK), and B cells. **Myeloid populations** encompass granulocytes (basophils, eosinophils, neutrophils), monocytes, macrophages, dendritic cells (DCs), and mast cells. Markers such as CD3, CD4, CD8, CD11b, CD14, CD19, CD56, and others are annotated with species-specific expression patterns (human-only, mouse-only, or both), strain-specific markers (str.-sp.), activation markers (activ.), degranulation markers (degr.), tumor necrosis factors (TNF), markers expressed during the immature stage of cell development (immat.), and specific markers for distinct subpopulations (e.g., pDCs and cDCs1).Particular attention is given to markers relevant for NK cell identification (e.g., NK1.1, CD56, NKG2, NKRP1, and KLRB1) and functional evaluation (e.g., CD107a, TRAIL, and FasL).
