# Supplementary material for: Translational Insights into NK Immunophenotyping: Comparative Surface Marker Analysis and Circulating Immune Cell Profiling in Cancer Immunotherapy
Source: Int J Mol Sci. 2025 Sep 30;26(19):9547. doi: 10.3390/ijms26199547 (PMC12524738; doi:10.3390/ijms26199547)
Supplement: Supplementary file 1 [file ijms-26-09547-s001.zip › Table S1. Relevance and limitations Table.pdf]

Supplementary Table S1. Relevance and limitations - Comparison of Key NK Cell Surface Markers

	Marker	Human NK Cells	C57BL/6 Mouse NK Cells	BALB/c Mouse NK Cells	Primary Functional Association	Translational Considerations & Limitations
**Pan-NK Identification**	CD56	Definitive marker ($CD3^{-}CD56^{+}$); subtypes: $CD56^{dim}$ (cytotoxic) and $CD56^{bright}$ (regulatory)	Not expressed	Not expressed	Adhesion, subset definition	**Major limitation:** No mouse ortholog. Precludes direct phenotypic translation.
	NKp46	Expressed on most NK cells; also on rare T-cell subsets	Expressed ($CD3^{-}NKp46^{+}$); also on rare T-cell subsets	Expressed ($CD3^{-}NKp46^{+}$); primary identifier	Activating cytotoxicity receptor	**Conserved marker.** Useful for cross-species identification but not entirely NK-specific in either species.
	CD49b	Weakly expressed on circulating NK cells	Expressed; used in combination (e.g., $CD3^{-}CD49b^{+}$)	Expressed; used in combination (e.g., $CD3^{-}CD49b^{+}$)	Adhesion, migration	More reliable for mouse NK identification. Human circulating NK cells show weaker expression.
	NK1.1	Not applicable	Definitive marker ($CD3^{-}NK1.1^{+}$)	Not expressed	Activating receptor	**Strain-specific limitation.** Useless in BALB/c, NOD, and other common strains.
**Activation & Functional State**	NKG2D	A key activating receptor for almost all NK cells and some T cells	Key activating receptor; expressed by NK and some T cells	Key activating receptor; expressed by NK and some T cells	Recognition of "stress-induced" ligands (e.g., MICA/B, RAE-1)	**Functionally conserved.** An ideal translational marker for assessing NK cell effector potential. Ligands differ.<br>**Limitation:** Not NK-specific, presented in other cytotoxic cells.
	CD16	High expression on $CD56^{dim}$ subset; mediates ADCC	Expressed (FcγRIII); homology closer to human CD32A	Expressed (FcγRIII)	Antibody-Dependent Cellular Cytotoxicity (ADCC)	**Functional homology but structural divergence.** Regulatory mechanisms (e.g., shedding) differ significantly.<br>**Limitation:** The specificity of the antibodies used is critically important due to the very high similarity.
	CD107a	Surface exposure upon degranulation	Surface exposure upon degranulation	Surface exposure upon degranulation	Marker of recent degranulation/cytotoxic activity	**Perfectly conserved functional marker.** Gold standard for measuring cytotoxic function across species.<br>**Limitation:** Not NK-specific, presented in other cytotoxic cells.
	CD69	Early activation antigen	Early activation antigen	Early activation antigen	Very early activation marker	**Conserved early activation marker.** Useful for assessing recent activation but not NK-specific.
**Inhibitory Receptors**	NKG2A	Inhibitory receptor (CD94/NKG2A); ICI target	Inhibitory receptor (CD94/NKG2A); binds Qa-1b	Inhibitory receptor (CD94/NKG2A); binds Qa-1b	MHC class I recognition; immune checkpoint	**Functionally conserved.** A promising target with translational potential for checkpoint inhibition.
	KIR Family	Diverse family of inhibitory/activating Ig-like receptors	Not present	Not present	MHC class I recognition; education/licensing	**Major species difference.** Fundamental difference in MHC recognition machinery limits translation of KIR-specific mechanisms.
	Ly49 Family	Not present	Diverse family of inhibitory/activating C-type lectin receptors (e.g., Ly49C, Ly49G)	Expressed (e.g., Ly49G); different repertoire than C57BL/6	MHC class I recognition; education/licensing	**Functional analog of KIRs, but structurally distinct.** Repertoire is highly strain-specific, complicating standardized panels.
